# Significantly Reducing Post-Tonsillectomy Haemorrhage Requiring Surgery by Suturing the Faucial Pillars: A Retrospective Analysis

**DOI:** 10.1371/journal.pone.0047874

**Published:** 2012-10-31

**Authors:** Götz Senska, Hilal Schröder, Carolin Pütter, Philipp Dost

**Affiliations:** 1 Hals-, Nasen-, Ohren-Heilkunde, Plastische Operationen, Marienhospital Gelsenkirchen GmbH, Gelsenkirchen, Germany; 2 Institut für Medizinische Informatik, Biometrie und Epidemiologie Universitätsklinikum Essen, Germany; UCL Institute of Child Health, University College London, United Kingdom

## Abstract

**Background:**

The tonsillectomy is one of the most frequently performed surgical procedures. Given the comparatively frequent postsurgical bleeding associated with this procedure, particular attention has been paid to reduction of the postoperative bleeding rate. In 2006, we introduced routine suturing of the faucial pillars at our clinic to reduce postoperative haemorrhage.

**Methods:**

Two groups from the years 2003–2005 (n = 1000) and 2007–2009 (n = 1000) have been compared. We included all patients who had an elective tonsillectomy due to a benign, non-acute inflammatory tonsil illness. In the years 2007–2009, we additionally sutured the faucial pillars after completing haemostasis. For primary haemostasis we used suture ligation and bipolar diathermy.

**Results:**

The rate of bleeding requiring second surgery for haemostasis was 3.6% in 2003–2005 but only 2.0% in 2007–2009 (absolute risk reduction 1.6% (95% CI 0.22%–2.45%, p = 0.04)). The median surgery time—including adenoidectomy and paracentesis surgery—increased from 25 to 31 minutes (p<0.01).

**Conclusions:**

We have been able to substantiate that suturing of the faucial pillars nearly halves the rate of postoperative haemorrhage. Surgery takes 8 minutes longer on average. Bleeding occurs later, mostly after 24 h. The limitations of this study relate to its retrospective character and all the potential biases related to observational studies.

## Introduction

Tonsillectomy is one of the most frequently performed surgical procedures [1].

Considering that it is an elective procedure, it is often connected with a comparatively high bleeding rate. Furthermore, bleeding in this location (the upper airways) always represents a significant risk. For this reason, there has been and still is a great deal of discussion regarding the different tonsillectomy techniques as well as primary haemostasis and the effect on reducing postoperative bleeding [2], [3].

In Germany, the rate of postoperative haemorrhage requiring second surgery after tonsillectomy is 3% [4]. However, a rate of less than one per cent of postsurgical bleeding can be found in the international literature [5], although comparison of these results is difficult because of imprecise definitions of haemorrhage and different patient collectives. For primary haemostasis, two different techniques are mainly used, bipolar diathermy and suture ligation [6], [5]. Suturing the faucial pillars is typically used in severe haemorrhage where other techniques have failed [7].

The technique of suturing the faucial pillars is used routinely as part of uvulopalatopharyngoplasty, after performing the tonsillectomy, with postsurgical haemorrhage occurring rarely. We therefore decided in 2006 to include this operative step in our normal, elective tonsillectomy procedures.

To obtain information on whether suturing of the faucial pillars leads to a decrease in the postoperative haemorrhage rate, we performed a retrospective data analysis.

## Methods

We analysed the data of patients in both comparison groups (with and without suturing the faucial pillars) who underwent tonsillectomy in the years 2003–2005 and 2007–2009. To exclude the stage of adjustment to the new operative technique, we did not analyse data from 2006.

We included in each group of the study 1000 patients who had electively undergone surgery for chronic or recurrent tonsillitis or hyperplasia of the tonsils. We evaluated 1000 patient records working back from December 2005 and 1000 patient records working forwards from January 2007. We decided to analyse 1000 records each after checking 400 patients in a first step. We saw a strong effect, but no significance. After a power calculation of sample size we decided to analyse 1000 records in each cohort.

We included every documented, even minor, haemorrhage. As such we also evaluated bleeding that was reported by the patient or the staff without verification by a medical doctor.

We defined primary haemorrhage as a bleeding within 24 h hours after surgery, all later occurring bleeding as secondary haemorrhage.

We also included patients who underwent other surgery during the same operation, as for example adenoidectomy.

With regard to postoperative haemorrhage, we analysed only tonsillectomy bleeding. If a patient underwent an adenotonsillectomy and bled due to the adenoidectomy wound, we did not take this haemorrhage into account for this study.

We retrospectively analysed 87 patient records from 2003, 485 records from 2004, 428 records from 2005, 340 from 2007, 382 from 2008 and 278 from 2009. This gave us two patient cohorts, each with 1000 records for comparison, who had been operated on with and without suturing the faucial pillars.

The operations were performed mostly by residents under the supervision of a senior physician. All operations were performed under general anaesthesia and on a lying patient with reclined head.

The tonsillectomy technique was the same in all years: after cutting the mucosa of the anterior tonsil pillar down to the root of tongue and medial to the uvula with scissors, the superior end of the tonsil was exposed. The tonsil was then freed extracapsularly using a surgical rasp. When indicated, the tonsil was cut from the root of the tongue using a wire sling.

For haemostasis during tonsillectomy as well as haemostasis after returnement to the operation theatre we used suture ligation in all years. The use of bipolar diathermy was reserved for experienced surgeons and in subordinated manner. We never used topically added substances or infiltration of adrenalin.

In the years 2007–2009, we additionally sutured the faucial pillars on both sides with two chain-block sutures after completing haemostasis.

In case of failing conservative methods for haemostasis (i.e. ice water, waiting for spontaneous haemostasis) patients were returned to the operation theatre. We never changed this standard over the years, respectively both cohorts. The decision to return a patient to the operation theatre was made the same over the years.

Normally the patients stayed in hospital until the fifth (SD 0.99) postoperative day.

The data was evaluated using the hospital information system, according to which patient records were checked and the data verified and completed. Since using the new method, we have controlled the haemorrhage rates by analysing the number of readmissions as well as surgery due to postoperative tonsillectomy haemorrhage by checking the data of our hospital controlling centre.

### Statistical methods

We used standard descriptive to display sample characteristics. Differences of count data between patients investigated in the years 2003 to 2005 and patients investigated in 2007 to 2009 were tested using Fisher's exact test or, where appropriate (cell counts in cross table >5), using the Chi2-test. In addition, we used conditional logistic regression to address the clustering of surgeries within surgeons and to address effects of experience. These analyses were limited to those surgeons who contributed to both patient cohorts. Continuous variables were analysed by the non-parametric Mann-Whitney test, since we cannot assume the normal distribution for the data. Effect size estimators are presented with 95% confidence intervals (95% CI) and all reported p-values are explorative, two-sided and nominal (,i.e. not adjusted for multiple testing). The level of significance α for each test was 0.05 (two-sided).

### Ethic statement

The datas were analysed after approval by the ethics committee of the Ärztekammer Westfalen Lippe and the Westfälische Wilhelms University Münster Germany. We did not obtain informed consent from the patient due to a statement of this committee, that analysing patient data retrospectively requires no informed consent according to the Gesundheitsdatenschutzgesetz Nordrhein Westfalen (Health Data Protection Act of the German State Nordrhein Westfalen).

## Results

445 of the 1000 patients from the years 2003–2005 were male, 555 female. In the years 2007–2009, 428 of the 1000 patients were male, 572 female. There was no significant difference between the sex distribution in the samples (p = 0.47).

The average age was 17 years in 2003–2005, in 2007–2009 16 years (2003–2005: minimum: 1 year, maximum 77 years; 2007: minimum: 2 years, maximum 77 years) Over the half of the patients were under 16 years old (n = 1051; 52.5%).

The operations were performed by 29 different surgeons. 10 surgeons did both techniques. Of the operations done by the 10 surgeons 384 took place in 2003–2005 and 710 in 2007–2009.

In 2003–2005 200 patients have been under five years old, in 2007–2009 211. Between five and 16 years old have been 351 patients in 2003–2005 and 350 in 2007–2009. Over 16 years old have been 449 in 2003–2005 and 439 in 2007–2009.

The median surgery time was 25 (lower (Q1) - upper quartile (Q3): 19–33) minutes in 2003–2005, in 2007–2009 31 (Q1–Q3: 24–40) minutes, which equates to an average increase of 30% (p = 0.01).

Postoperative haemorrhage differed between the two samples: in 2003–2005, without suturing the faucial pillars, 86 (8.6%) had a bleeding event, 36 (41.9%) of whom had to be returned to the operating theatre.

In 2007–2009, the number of patients who suffered post-tonsillectomy haemorrhage was 66 (6.6%), 20 (30.3%) of whom had to be returned to the operating theatre.

In 2003–2005 5 patients who had to be returned to the operating theatre have been under five years old, in 2007–2009 2. Between five and 16 years old have been 10 patients in 2003–2005 and 5 in 2007–2009. Over 16 years old have been 21 in 2003–2005 and 13 in 2007–2009. There is no significant difference between the cohorts (p = 0,867).

The frequency of overall haemorrhage did not differ significantly between the samples (OR = 0.75 (95% CI 0.53–1.06), p = 0.11), whereas there was a significant difference in the frequency of bleeding events requiring a return to the operating theatre, with an odds ratio of 0.55 (95% CI 0.30–0.98) and a p = 0.04.

The same trend can be seen if the clustering within surgeons who did both techniques is taken into account (overall haemorrhage: OR = 0.70 (95% CI 0.42–1.17) p = 0.17; returned to theatre: OR = 0.80 (95% CI 0.33–1.94) p = 0.620; for all surgeons the results were very similar).

In terms of an absolute risk reduction an odds ratio of 0.55 for bleeding events requiring a return to the operating theatre translates into 1.60% (95% CI 0.22%–2.45%) or a number needed to treat of 63 patients.

The time of occurrence of haemorrhage differed significantly (p<0.001) between both samples. In 2003–2005 haemorrhage took place on the third (SD 3.5) postoperative day on average; in 2007–2009 on the fifth postoperative day (SD 4.1).

**Figure 1:**
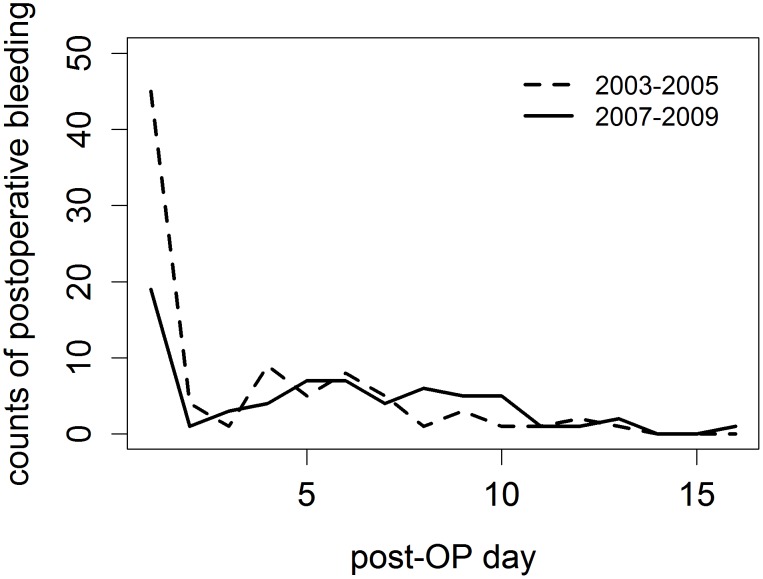
Onset of overall haemorrhage.

Furthermore, a significant difference was shown regarding the point of time of postoperative bleeding that required haemostasis in the operating theatre (p<0.001). In 2003–2005 this was on average on the second postoperative day (SD 3.1), in 2007–2009 on the sixth day (SD 1.4). The median of days of postoperative haemorrhage requiring a return to the operating theatre was day one in 2003–2005. In 2007–2009 it was day five. In 2003–2005, 20 of the 36 (55.5%) haemorrhages requiring management in the operating theatre occurred within the first 24 h. In 2007–2009 that number was 4 out of 20 (25%) haemorrhages (Figure 1).

The number of postoperative haemorrhages within 24 h that had to be managed in the operating theatre differed significantly between the two patient cohorts (p = 0.01). The latest onset of haemorrhage requiring a return to the operating theatre was on day 12 in 2003–2005 and on day 13 in 2007–2009.

Amongst those patients who experienced haemorrhage in 2003–2005, 29% (n = 25) had already been discharged. In 2007–2009, every second patient (n = 32) had been discharged. Normally our patients are discharged on the fifth postoperative day. In 2003–2005, 7 of the 25 readmitted patients had to be returned to theatre (28%). In 2007–2009, 10 of the 32 readmitted patients (31%) had to be returned to theatre. This represents no significant difference (p = 0.98).

Table 1 describes detailed information to descriptive statistics of the two patient cohorts.

**Table 1:** Descriptive statistics of the two patient cohorts (n = 1000 each).

	2003–2005	2007–2009	p-Value	odds ratio	95% CI for odds ratio
**Patients**	n = 1000	n = 1000			
**Overall haemorrhage**	86	66			
% of patients	8.6	6.6	0.1	0.75	(0.53; 1.06)
**Within 24 h**	36	17			
% of haemorrhage	41.9	25.8	0.06	0.48	(0.22; 1.02)
% of patients	3.6	1.7	0.01	0.46	(0.24; 0.85)
Within 48 h	37	20			
% of haemorrhage	43.0	30.3	0.15	0.58	(0.28; 1.19)
% of patients	3.7	2-0	0.03	0.53	(0.29; 0.95)
**Returned to theatre**	36	20			
% of patients	3.6	2	0.04	0.55	(0.30; 0.98)
% of “overall haemorrhage”	41.9	30.3	0.19	0.61	(0.29; 1.25)
**Within 24 h**	20	4			
% of patients	2	0.4	0.001	0.20	(0.05; 0.59)
% of returned to theatre	55.6	20	0.01	0.21	(0.04; 0.81)
% of “overall haemorrhage”	23.3	6.1	0.001	0.21	(0.05; 0.69)
Within 48 h	23	4			
% of patients	2.3	0.4	0.0002	0.17	(0.04; 0.50)
% of returned to theatre	63.9	20	0.002	0.15	(0.03; 0.58)
% of “overall haemorrhage”	26.7	6.1	0.001	0.18	(0.04; 0.56)
**Day of onset of haemorrhage**	3	5.2	<0.001		
**Patients already discharged**	25	32			
% of haemorrhage	29.1	48.5	0.02	2.27	(1.11; 4.76)
% of patients	2.5	3.2	0.42	1.28	(0.74; 2.27)
**Returned to theatre**	7	10			
% of already discharged	28	31.3	0.98	1.16	(0.32; 4.35)

Neither in 2003–2005 nor in 2007–2009 was it necessary for a patient to be returned to theatre twice. Likewise blood transfusions or opening the neck for haemostasis, for example by ligation of the external carotid artery, were not necessary at any time.

Furthermore we never saw any infection of the sutured faucial area.

Finally, we also explored the impact of experience (years of work as surgeon) on haemorrhage. We observed no evidence for an effect on both overall haemorrhage (OR per year 0.94, p = 0.41) and on haemorrhage that led to a return to the operation theatre (OR per year 0.97, p = 0.81).

## Discussion

The sex distribution after tonsillectomy in favour of female patients corresponds to the group of all patients that underwent tonsillectomy in Germany and Great Britain [4], [5]. The overall age distribution as well as the age distribution of the patients returned to the operation theatre is likewise similar to the aforementioned studies. As is known, especially children and teenagers undergo tonsillectomy due to recurrent or chronic tonsillitis.

The mean operating time in both patient cohorts was around half an hour, but we saw a significant difference of eight minutes. The time required to suture the faucial pillars after tonsillectomy is therefore of some significance. The rate of haemorrhage leading to a return to the operating theatre was significantly reduced in 2007–2009 compared to 2003–2005. A similar direction of effect was observable if the clustering of operations within surgeons who did both techniques was taken into account.

We saw no significant reduction of overall bleeding. This is no statistical effect due to inadequate power. This might be due to an observational bias after the decision to suture the faucial pillars. In light of the fact to use an additional method to the routinely used standard tonsillectomy surgery, we have been more aware of complications. We documented even stricter to control the effects. We have a standard procedure for returning a patient to the theatre, but no standard for documenting all bleeding episodes. In the latter cohort we documented even every anamnestic bleeding that could not be objectified.

It is difficult to compare this study with other studies on this topic directly. This is mainly because the definitions of primary versus secondary haemorrhage and the necessity to return to the operating theatre are vastly different. Nevertheless, there are numerous studies that deal with this subject in a similar manner.

We defined primary haemorrhage as a bleeding within 24 h hours after surgery, all later occurring bleeding as secondary haemorrhage.

Overall there is a huge variety of data regarding the rate of postoperative haemorrhage after tonsillectomy that led to a return to the theatre. They range from under one and up to six per cent [5], [8], [9], [10]. The Audit of British Association of Otorhinolaryngologists published a notably low rate of haemorrhage that led to a return to theatre. They saw around one per cent of bleedings in 40,000 tonsillectomies [5]. Such low rates were questioned until just a few years ago by Windfuhr et al. for their imprecise study design and short follow up [11]. Now, however, the publication of the British audit removes any such doubt as far as the described surgery and documentation are concerned. At the same time, a German audit by the BQS (German Institute for Quality and Patient Security) found a bleeding rate of 3.1% requiring a return to the theatre in 116,000 tonsillectomies [4]. These findings coincide with data from other studies that analysed their own patients or took an audit [12], [13],[14], [15]. Our rate of 2% of postoperative haemorrhage managed in the operating theatre is among the lowest ever published. The relative risk reduction of 44% enabled us to nearly halve all haemorrhage treated in the operating theatre.

The onset of bleeding coincides in 2003–2005 with the published data in the international literature. This means that most haemorrhage leading to a return to the theatre occurs with a likelihood of 60–70% as a primary haemorrhage within the first 24 h [9], [15], [16]. However, in our sample between 2007 and 2009, nearly all haemorrhage occurred later than 24 h after surgery. Although early onset of bleeding is known to be a consequence of poor quality surgery and more hazardous than secondary bleeding [4], [13], [12], it must be taken into consideration especially with regard to German discharge management. One in two patients who returned to theatre in 2007–2009 had already been discharged. At the same time, none of the haemorrhage was so extreme that we considered abandoning the additional method. Contrary to other studies, we saw no fatal bleedings caused by arterial dissection or aneurysma spurium [16], [17], [18], and haematomas were observed rarely [19]. Moreover, the very few cases of haematomas needed no surgical intervention and had no negative influence on patient comfort. Permanent nasal regurgitation never occurred. At other institutions such complications led them to abandon methods such as suture ligation completely in favour of bipolar diathermy or suturing in the caudal tonsil pillar region only [17], [18]. Likewise, we saw no haematomas requiring surgery as described in a study evaluating the benefits of suturing the faucial pillars for pain reduction [20].

In summary, we are convinced that by additionally suturing the faucial pillars we produce a benefit for our patients by reducing haemorrhage, especially haemorrhage requiring additional surgery. Despite the later onset of bleeding, we see no disadvantage for the patients. We saw no nasal regurgitation or increased need for analgetics, although the subject of pain will be dealt with in a further study.

This study has some possible limitations. Firstly, our study compares the retrospective data of two samples with and without suturing the faucial pillars. Secondly, we are not able to know whether patients were readmitted to other hospitals, which is a problem common to most of the studies. Thirdly, this study could be affected by all possible biases related to observations research – e.g. observation bias as every surgeon, nurse and doctor knew about the additional suturing. However, to address this problem to some extent we did not include data on 2006, the year in which we introduced the new procedure.

Therefore, we see no reason based on our experience to withhold this technique from patients before being explored in e.g. phase III randomized controlled trials.

## Conclusion

Suturing the faucial pillars leads to a significant reduction in postoperative haemorrhage requiring additional surgery, while operating time increases by around 30%. The onset of bleeding was delayed.
